# Effects of Carbohydrate Intake on *Anopheles darlingi* and *Anopheles deaneorum* Fitness under Lab-Reared Conditions

**DOI:** 10.3390/insects15040240

**Published:** 2024-03-30

**Authors:** Najara A. C. Santos, Mirilene M. Martins, Alice O. Andrade, Alessandra S. Bastos, José D. C. Pontual, Jéssica E. Araújo, Marina L. Rocha, Jansen F. Medeiros, Maisa S. Araujo

**Affiliations:** 1 Plataforma de Produção e Infecção de Vetores da Malária (PIVEM), Laboratório de Entomologia, Fiocruz Rondônia, Porto Velho 76812-245, RO, Brazil; najara.akira@fiocruz.br (N.A.C.S.); alice.oandrade@fiocruz.br (A.O.A.); alessandra.bastos@fiocruz.br (A.S.B.); jose.pontual@fiocruz.br (J.D.C.P.); jessica.evangelista@fiocruz.br (J.E.A.); marina.rocha@fiocruz.br (M.L.R.); jansen.medeiro@fiocruz.br (J.F.M.); 2 Instituto Nacional de Epidemiologia da Amazônia Ocidental (INCT-EpiAMO), Porto Velho 76812-245, RO, Brazil; 3 Programa de Pós-Graduação em Biologia Experimental, Fundação Universidade Federal de Rondônia, Fiocruz Rondônia, Porto Velho 76812-245, RO, Brazil; mirimendes53@gmail.com; 4 Programa de Pós-Graduação em Saúde Pública, Faculdade de Saúde Pública, Universidade Federal de São Paulo, São Paulo 01246-904, SP, Brazil; 5 Programa de Pós-Graduação em Conservação e uso de Recursos Naturais—PPGReN, Fundação Universidade Federal de Rondônia, Porto Velho 76812-245, RO, Brazil; 6 Laboratório de Pesquisa Translacional e Clínica, Centro de Pesquisa em Medicina Tropical (CEPEM), Porto Velho 76812-329, RO, Brazil

**Keywords:** nutrition, sugar meal, malaria vector, colonization

## Abstract

**Simple Summary:**

The carbohydrate source is a nutritional aspect critical for the basic behaviors of mosquitoes, such as flying and mating. For the maintenance of a colony of mosquitoes, it is important to choose a good source of carbohydrates that increases mosquito fitness. In this study, we found that a 15% honey solution increased mosquito longevity in comparison with a 10% sugar solution, probably due to the nutritional value of honey. Accordingly, a carbohydrate source based on a 15% honey solution could be a better choice for maintenance of *Anopheles darlingi* and *Anopheles deaneorum* in the lab-rearing context.

**Abstract:**

The maintenance of a highly productive colony of anopheline mosquitoes requires standardized methods in order to obtain a sufficient number of homogeneous individuals for malaria research. In this context, nutritional status may affect survival, fecundity, and the capacity to support pathogen development. Here we assess the effects of carbohydrate sources on fecundity, survival, and susceptibility to *Plasmodium vivax* infection in colonies of *Anopheles darlingi* and *Anopheles deaneorum* mosquitoes. Newly emerged females from each species were fed either 10% sugar or 15% honey solutions until the end of each experiment. The type of carbohydrate meal did not impact any entomological parameters for *An. deaneorum*, except for survival. For both species, honey meal significantly increased median survival post-emergence by three to four days, probably due to its nutritional value. For *An. darlingi* fed with honey, a higher mean frequency in stage 5 was observed at 48 h post-blood-meal, which could indicate a delay in the digestion process. However, no effects on fecundity parameters were observed. Regarding susceptibility, *An. darlingi* fed with sugar exhibited a low intensity of sporozoites, although any negative effects of sucrose on sporozoites invasions in the salivary glands are unknown. Based on the increase in mosquito survival, a carbohydrate source composed of 15% honey solution could be better for maintaining *An. darlingi* and *An. deaneorum* in the lab-rearing context.

## 1. Introduction

The rearing and maintenance of anopheline colonies in the laboratory is a prerequisite for understanding various aspects of mosquito biology, insecticide resistance, and pathogen–microbiota–mosquito interactions, as well as for studying and assessing transmission-blocking activities of drugs and vaccines [1]. To study the interaction between *Anopheles* mosquitoes and malaria vivax, we have colonized two species of *Anopheles* in our laboratory: *Anopheles darlingi* [2] and *Anopheles deaneorum* [3].

*Anopheles darlingi* is the main malaria vector for both *Plasmodium vivax* and *Plasmodium falciparum* in deforested regions of the Amazon biome [4]. The second species, *An. deaneorum*, belongs to the Albitarsis complex and remains a secondary vector for malaria transmission in certain regions of the Western Amazon [5,6].

Establishing anopheline colonies presented a challenge due the species’ limited ability to mate in restricted spaces [7]. Currently, free-mating laboratory populations of *An. darlingi* have been established to enhance studies on malaria transmission in Neotropical anopheline mosquitoes [2,8,9,10]. However, maintaining a highly productive colony is a labor-intensive effort that typically requires standardized rearing methods to obtain a sufficient number of homogeneous and susceptible mosquitoes for the experimental procedures [8,11]. In this context, nutritional status plays a crucial role in producing healthy adult mosquitoes with the energy necessary for survival [12,13,14] and fecundity [12,15], and capable of supporting pathogen replication, development, and transmission [16,17].

In nature, adult mosquitoes primarily ingest plant-derived sugars [15] to meet their physiological needs, e.g., for males to perform swarming behavior [18] and for females to fly in search of mates, blood-meal sources, and oviposition sites [14,15]. This is important not only for wild mosquitoes but also for those reared in laboratory conditions, where maintaining high adult vitality and mating ability is essential for colony maintenance [11]. In research laboratories, adult anopheline mosquitoes are typically fed a 10% sugar solution in the contexts of rearing [9,10,19,20,21,22] and maintaining experimental *Plasmodium*-infected mosquito species [9,23,24]. However, other carbohydrate sources and varying concentrations can also be found in the literature, such as 5% glucose solution, 10% dextrose solution, 5% sucrose solution, 8% honey–water solution, and 10% honey–water solution [8,25,26,27].

During the initial establishment of our colonies of *An. darlingi* and *An. deaneorum* [2,3], a high mortality rate for males had been observed that limited our attempts to induce copulation using flashes of light. The introduction of honey as a carbohydrate source improved male survival. At that time, the concentration of the 15% honey solution was determined based on the viscosity of the available honey. Honey produced by stingless bees tends to be more liquid in consistency. Therefore, the establishment of our highly productive *An. darlingi* and *An. deaneorum* colonies was achieved using a 15% honey solution sourced from local honey produced by stingless bees [2,3]. After obtaining these colonies, we sought to investigate whether maintaining mosquito colony with a 15% honey solution could interfere with certain aspects of *An. darlingi* and *An. deaneorum* mosquitoes, particularly with regard to their susceptibility to *Plasmodium* infection.

Our hypothesis was that a carbohydrate meal based on a 15% honey solution would enhance the fecundity, survival, and susceptibility to *P. vivax* in mosquitoes, compared to a common 10% sugar solution, which is the standard carbohydrate source for mosquito colonies [9,10,19,20,21,22]. Here, we present our findings on the effects of different carbohydrate sources on biological parameters related to colony maintenance, including blood digestion, fecundity, male and female survival, and the susceptibility of laboratory-reared females from both the *An. darlingi* and *An. deaneorum* species to *P. vivax* infection.

## 2. Materials and Methods

### 2.1. Mosquito Rearing

All experiments were performed using laboratory-reared mosquitoes from the Production and Infection of Malaria Vectors Platform (PIVEM/Fiocruz RO) in Brazil. Colonies of *An. darlingi* and *An. deaneorum* have been established and maintained since 2018 by the principal authors of Araujo et al. [2] and Araujo et al. [3]. The colonies are reared under controlled conditions of temperature (26 ± 1 °C) and humidity (70% ± 10%), and kept under a 12 h light–dark cycle. Adult mosquitoes are fed with a 15% honey solution *ad libitum*, and rabbit blood has been used to maintain egg production across generations. The use of rabbits for maintenance and experimental studies was approved by the Animal Utilization Ethical Committee of Fiocruz Rondônia (CEUA) under protocol 2019/10.

Larvae have been reared in plastic containers filled with distillated water (1 L) which has been changed twice a week, and have been fed daily with TetraMin^®^ Marine fish food (Tetra GmbH, Melle, Germany).

To assess the effects of different carbohydrate sources on mosquitoes, all experiments were performed using newly emerged females. These females were separated into two experimental groups: (i) a group fed with 15% honey solution (local honey producer PROVE Vilhena/Rondônia/Brazil) *ad libitum*, referred to as the honey group; and (ii) a second group fed with 10% sugar solution (cane sugar, Barralcol, Barra do Bugres, Brazil) *ad libitum*, referred to as the sugar group. These carbohydrate sources were provided daily to their respective mosquito groups until the end of each experiment.

### 2.2. Blood Digestion

To evaluate the effects of honey and sugar feeding on blood digestion, females aged 5–6 days were separated from each experimental group and allowed to feed on physically restrained rabbits for 20 min. Twenty (*n* = 20) fully engorged females were placed individually in Falcon tubes (50 mL) adapted with tulle lids. The gonotrophic cycle/blood digestion of each female was observed at 24 h, 48 h, 72 h, and 96 h, following the Sella scale, as described by Detinova et al. [28], to classify the degree of blood-meal digestion in categories ranging from fully engorged females (stage 2) to complete blood digestion (stage 7). Honey or sugar solution was available ad libitum until the end of the experiment. Each engorged female was considered a biological unit of replication, and the entire experiment was repeated three times on different days.

### 2.3. Fecundity

To investigate whether different carbohydrate sources have varying effects on mosquito fecundity, we assessed the engorgement rate, egg production rate (eggs/female), and hatching rate. For this purpose, 100 females, aged 5–6 days, from each experimental group were placed in a new cage and allowed to feed on rabbit for 20 min. Only fully engorged females were transferred to a new cage, and their respective carbohydrate sources were provided *ad libitum*.

Two days after the blood meal, dark cups with filter paper and 1/3 filled with distilled water were placed inside the cage with the blood-fed gravid females. Mosquito females were allowed to oviposit for three days. The eggs were counted using a stereomicroscope (Leica EZ4, Leica Microsystems, Heerbrugg, Switzerland). After recording the egg count, the eggs were transferred to pans (30.3 × 22.1 × 7.5 cm) with filter paper and 1 L of distillated water for egg hatching. The eggs were kept in the pans for five consecutive days. On the fifth day, all larvae were counted and recorded to assess the egg hatching rate for each experimental group.

The biological unit of replication for engorgement rate and egg production was the cage containing the engorged females, while for the hatching rate, it was the pans. Two replications were performed per experimental group, and the entire experiment was repeated four times on different days.

### 2.4. Survival

To assess the effects of different carbohydrate sources on the lifespan of mosquitoes, 50 newly emerged males and 50 newly emerged females from each carbohydrate source group were monitored daily until the death of the last mosquito in the cage. Either 10% sugar or 15% honey solutions were provided ad libitum throughout the experiment for their respective experimental groups. Daily mortality was recorded for survival analysis. The experimental groups were conducted with three technical replications, and the entire experiment was repeated five and four times for *An. darlingi* and *An. deaneorum*, respectively, on different days.

### 2.5. Mosquito Infection

Artificial infections using a Direct Membrane Feeding Assay (DMFA) with blood from patients positive for *P. vivax* were performed on mosquitoes fed with sugar or honey solution to assess the effect of carbohydrate source on *P. vivax* infection in *An. darlingi* and *An. deaneorum*.

Blood collection from *P. vivax*-positive patients was performed at the Centro de Pesquisa em Medicina Tropical (CEPEM), in Porto Velho, Rondonia, Brazil. The blood collection was approved by Centro de Pesquisa em Medicina Tropical (CEPEM) Ethical Committee (protocol #28176720.9.0000.0011), and informed consent was obtained from the volunteers. Patients diagnosed with vivax malaria by Giemsa-stained blood smears were invited to participate in the study, with the following criteria: age > 18 years, not an indigenous person, not a pregnant woman, absence of signs or symptoms of severe malaria or concomitant disease, and formal agreement to the study’s procedures. Approximately 10 mL of blood was collected by venipuncture in heparinized Vacutainer tubes and stored at 37 °C until DMFA (within ~15 min).

Before DMFA, female mosquitoes from both species were deprived of their respective carbohydrate sources overnight. Two mL of infected blood from each patient were added to a 5 cm diameter glass membrane feeder fitted with a Parafilm membrane. The glass membrane feeders were accoupled in a water-bath to maintain a constant temperature of 37 °C. Cohorts of approximately 100 female mosquitoes from each species were selected, and each group fed on the infected blood in the glass membrane feeder for 30 min. Only fully fed mosquitoes were kept in the experiment aiming to assess the *P. vivax* sporogonic development. After the blood meal, the honey or sugar solution was provided every other day until the time of dissection. Daily mortalities were recorded from 1 to 14 days post-blood-feeding for survival analysis.

### 2.6. Mosquito Dissection

Around 20–30 mosquitoes were dissected on post-infection day 7 to record oocyst load in the midgut. The midguts were dissected in PBS 1× and stained with 0.2% commercial mercurochrome (SIGMA-ALDRICH, Steinheim, Germany). The presence of oocysts was determined under a microscope (10× magnification) (Leica ICC50W, Leica Microsystems, Heerbrugg, Switzerland). At 14 days post-infection (dpi), surviving mosquitoes were dissected to record sporozoite load in their salivary glands. The salivary glands of five individuals were pooled, ground with a glass tissue-grinder, and then centrifuged for 30 s. Sporozoite numbers were counted using a Neubauer chamber hematocytometer under a contrast microscope (40× magnification). Each mosquito was considered a biological unit of replication.

### 2.7. Statistical Analysis

To assess the effect of carbohydrate source on the blood digestion, we calculated the mean frequency of mosquitoes in each stage of gonotrophic cycle/blood digestion at various time points (24 h, 48 h, 72 h, and 96 h). The frequency of mosquitoes in each stage was compared between the honey and sugar groups, using unpaired t-tests. For fecundity, we estimated parameters including engorgement, number of eggs per female, and hatching rate. Data with normal distribution were compared using unpaired *t*-tests, while data with non-parametric distribution were compared using the Mann–Whitney test. Daily mortality data were used to construct Kaplan–Meier curves to conduct survival analysis with 95% confidence intervals, and the curves were compared using the log-rank test. The prevalence rate of oocyst in each group was compared using a chi-square test, while oocyst and sporozoite intensity were compared using the Mann-Whitney test. A *p*-value of less than 0.05 was considered statistically significant.

## 3. Results

We assessed whether different carbohydrate sources had different effects on lab-reared *An. darlingi* and *An. deaneorum* by observing blood digestion time, fecundity, survival, and infection parameters with *P. vivax*.

### 3.1. Blood Digestion

**Figure 1:**
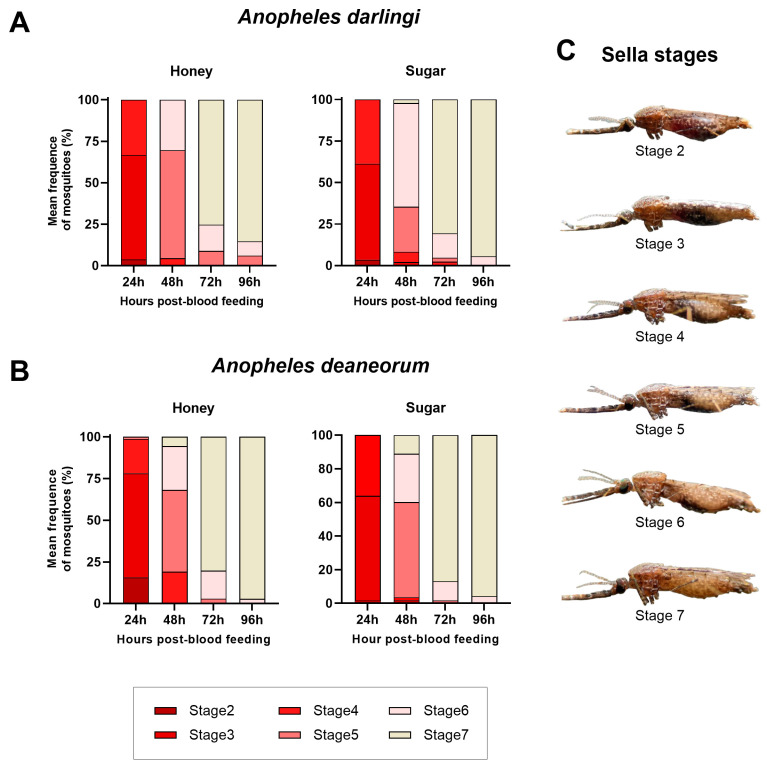
Frequency of blood digestion status of *Anopheles darlingi* and *Anopheles deaneorum* females under honey or sugar intake. Plots (**A**,**B**) show the mean frequency of each Sella degree observed in the females at each analyzed time point. (**C**) shows blood digestion stages according to Sella classification.

To evaluate the effects of different carbohydrate sources on blood feeding status, we classified the engorged females of *An. darlingi* and *An. deaneorum* according to Sella degrees at 24, 48, 72, and 96 h post-blood-meal. We observed that neither the 15% honey solution nor the 10% sugar solution changed the blood-feeding status of *An. darlingi* or *An. deaneorum* at the observed time points (Figure 1A,B and C; Table 1). The sole exception was *An. darlingi* fed with honey, which exhibited a higher mean frequency of females in stage 5 at 48 h post-blood-meal (hpb) (65.2%; SD ± 9.4; CI 95% 41.8–88.5%), compared to the sugar group (27%; SD ± 9.5; CI 95% 3.4–50.8) (*t*-test: t = 4.923; df = 3.999; *p* = 0.007).

### 3.2. Survival and Fecundity

**Figure 2:**
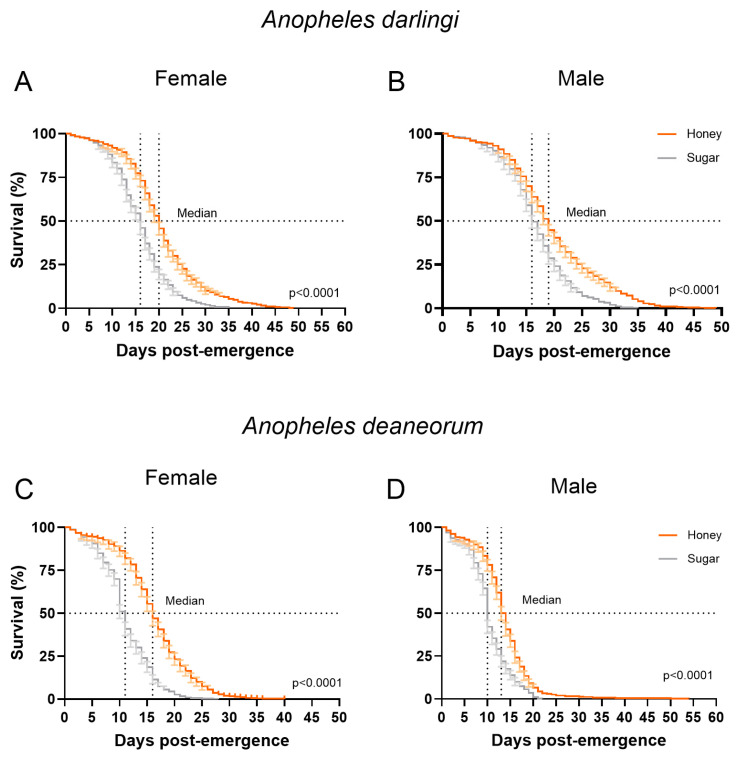
Survival curves of female and male *Anopheles darlingi* (**A**,**B**) and *Anopheles deaneorum* (**C**,**D**) mosquitoes fed with honey or sugar meal. Dotted lines indicate the median survival of each group. Error bars indicate 95% confidence interval.

Mosquitoes fed with honey solution showed increases in their lifespans compared to the sugar group (*p* > 0.0001). The honey solution resulted in an average increase of 13 days in lifespan for *An. darlingi* females and males, as well as for females of *An. deaneorum* (Figure 2A–C). For males of *An. deaneorum*, we observed an increase of 32 days in lifespan (Figure 2D). The median survival increased by 4 days for *An. darlingi* and *An. deaneorum* females (Figure 2A,C), while for males of both species, it increased by 3 days when mosquitoes were fed honey solution (Figure 2B,D).

However, there was no clear evidence indicating differences between the groups of *An. darlingi* and *An. deaneorum* fed with either honey or sugar solution as to the fecundity parameters (Table 2).

### 3.3. Mosquito Infection

To assess the performance of *An. darlingi* and *An. deaneorum* fed with either honey or sugar solution under *P. vivax* infection, we conducted six independent DMFA assays. The parasitemia of the *P. vivax* blood samples ranged from 1640 to 33,840 parasites/μL, and gametocytemia ranged from 40 to 1120 gametocyte/μL. Data from each DMFA is shown in Supplementary Table S1.

**Figure 3:**
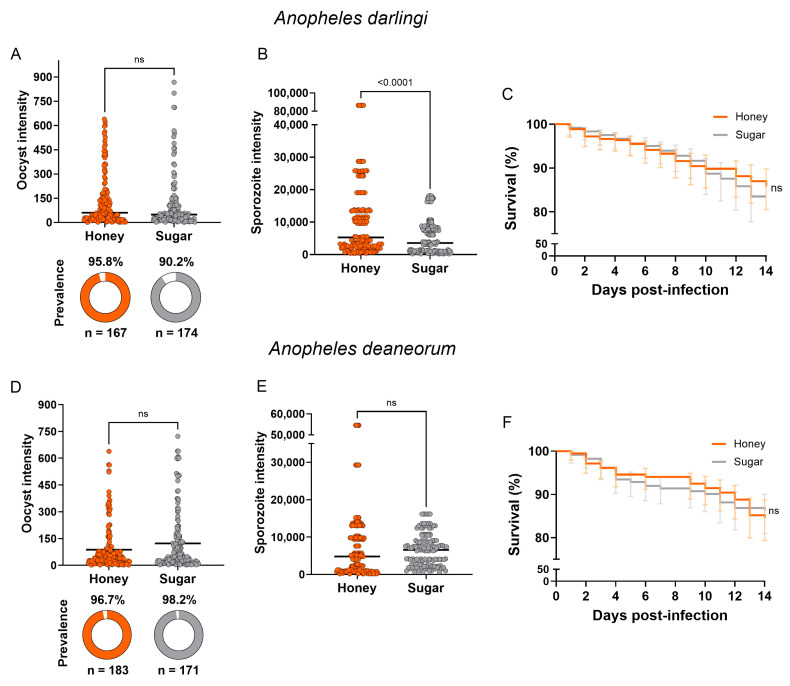
Infection parameters according to carbohydrate source. (**A**,**D**) show oocyst intensities of *Plasmodium vivax* experimental infection in *Anopheles darlingi* and *Anopheles deaneorum*, respectively. (**B**,**E**) show sporozoite intensities for each species according to carbohydrate source. (**C**,**F**) show survival curve post-infection for each species according to carbohydrate source. Bars indicate median, and “ns” indicates a statistical non-difference. Error bars indicate 95% confidence intervals.

*Anopheles darlingi* fed with honey solution showed an increase of 6.18% in the prevalence of infection (95.81%; CI 95% 91.4–98.1%) compared to *An. darlingi* fed with sugar solution (90.23%; CI 95% 84.8–93.8%) (Figure 3A). However, we did not observe clear statistical effects of the specific carbohydrate tested on the prevalence of infection for *An. darlingi* (χ^2^ = 3245; df = 1; *p*-value = 0.071). Increases in oocyst and sporozoite intensities were observed in *An. darlingi* fed with honey solution. Mosquitoes fed with honey showed a 22% increase in the intensity of oocyst (60; CI 95% 47–86) compared to mosquitoes fed with sugar solution (49; CI 95% 38–57) (Figure 3A); however, the effect was statistically unclear (U = 11,029; *p* = 0.060).

As for sporozoite intensity, the honey group achieved a median of 5280 sporozoites (CI 95% 4160–10,160), while the sugar group had a median of 3577 sporozoites (CI 95% 2933–6080) (Figure 3B). Sporozoite intensity, comparing the honey group and mosquitoes fed with sugar solution, was 47.6% higher in the honey group. Although the oocyst intensity distribution in the honey group did not show a statistically significant difference compared to the sugar group, the difference in sporozoite intensity between the groups was statistically significant (U = 7677; *p*-value < 0.0001).

On the other hand, for *An. deaneorum* mosquitoes, no differences were observed between the experimental groups. The prevalence of infection did not show a clear difference between the experimental groups based on carbohydrate source (χ^2^ = 0.06801, df = 1; *p*-value = 0.794) (Figure 3D). Although the oocyst intensity increased from 42 (CI 95% 68.6–105.9) in the honey group to 46.5 (CI 95% 98.4–147.2) in the sugar group, the difference was not statistically clear (U = 13,558, *p*-value 0.1338) (Figure 3D). No clear difference was observed in sporozoite intensity (U = 9049; *p*-value = 0.079), with the sugar group achieving a median of 6560 sporozoites (CI 95% 5880–7373) and the honey group 4800 sporozoites (CI 95% 6074–9328) (Figure 3E).

Finally, we assessed the effect of the carbohydrate source on the survival of *P. vivax*-infected mosquitoes. For both species, the survival analyses did not show clear differences between the survival curves of the experimental groups (*An. darlingi*: *p*-value = 0.750; *An. deaneorum*: *p*-value = 0.760) (Figure 3C,F).

## 4. Discussion

In laboratory-rearing conditions, we assessed the differences between two carbohydrate sources in some aspects of *An. darlingi* and *An. deaneorum* fitness. Our investigation found that honey and sugar solutions did not clearly show differences in terms of fecundity or susceptibility to *Plasmodium*. However, we observed a delay in blood digestion at 48 h post-blood-meal in the honey group, along with a substantial increase in both male and female longevity.

Based on a study that registered that honey compounds inhibit proteinases in honeybees [29,30], it was observed that a 10% concentration honey solution inhibited the trypsin enzyme in two mosquito species, *Aedes aegypti* and *Culex pipiens quinquefasciatus*, as well as bovine trypsin. Additionally, the authors found that ascorbic acid and riboflavin, both present in honey, had high inhibitory effects on trypsin. Trypsin enzymes play a role in blood digestion in mosquitoes, specifically in the hydrolysis of hemoglobin [31]. Mosquitoes in general store sugar meals in the crop before digestion, so proteinase inhibitors may remain away from the blood-digestion site in the midgut [30]. However, *An. darlingi* appear to quickly release the carbohydrate meal from the crop [32,33]. The high proportion of mosquitoes fed with honey solution found to be in stage 5 of blood digestion may be due to the presence of proteinase inhibitors in honey.

Despite the potential delay in blood digestion mentioned earlier, fecundity parameters assessed in the subsequent experiment were not affected by the different carbohydrate sources. This suggested that the observed difference in blood digestion may not have had a significant biological impact on the fecundity of the mosquitoes. Similarly, maintaining mosquitoes with either sugar or honey did not alter fertility measures. Sugar meals are known to increase glycogen and triglyceride reserves in females, which may contribute to oocyte development and improve female longevity after oviposition [34]. Although we did not assess the nutritional aspects and caloric values of honey and sugar solutions, both appear to be equally effective in promoting female fecundity. Further investigation is needed to understand the physiological and molecular aspects of digestion in *An. darlingi* and *An. deaneorum*, as well as the implications of diet on the reproduction of these neotropical anophelines.

It is well-documented that sugar meals increase the survival rates of mosquitoes [14,16,35,36,37]. In our study, mosquitoes fed with honey solution showed an increased lifespan compared to those fed with sugar solution. The nutritional value of honey solution may be the reason for this improvement in the longevity of *An. darlingi* and *An. deaneorum*. Honey is known to contain carbohydrates and amino acids, and the presence of amino acids in the sugar feeding of mosquitoes may enhance their survival, as observed in *Culex quinquefasciatus* [13].

In the context of mosquito infection, the diet is a key regulator for the immune system, working against pathogen establishment. Almire et al. [38] describe a high expression of antiviral genes mediated by a sugar meal rich in glucose and fructose in *Ae. aegypti* mosquitoes. In anopheline mosquitoes, the efficiency of the immune response via melanization against *Plasmodium* infection was found to be worse in mosquitoes fed with a lower-sugar concentrate. This suggests that blood meals and sugar feeding are important for mounting an efficient melanization response in *An. stephensi* [39]. Furthermore, *Plasmodium* infection has been shown to increase sugar feeding in anopheline mosquitoes. This observed phenomenon could benefit the parasite by reducing mosquito mortality rates or even ensuring a high glucose supply for oocyst development [40,41]. The results for *P. vivax* infection suggest that sugar meals based on honey or sugar solutions may not interfere with *P. vivax* development, and that both carbohydrate sources can be used during mosquito maintenance throughout the sporogonic cycle. Although mosquitoes fed with sugar meals showed lower sporozoite intensity than those fed with honey, there are no indications that sucrose affects sporozoite invasions in the salivary glands [42].

## 5. Conclusions

The comparison between honey and sugar solutions revealed no significant differences in fecundity parameters, which are crucial for colony propagation. However, a notable increase in the longevity of both female and male mosquitoes fed with honey suggests its potential as an alternative for anopheline rearing in laboratory settings. Importantly, honey solution did not compromise mosquito susceptibility to *Plasmodium*, which is vital for studies assessing the transmission-blocking effectiveness of compounds. Considering the potential challenges in obtaining or affording pure honey, using sugar meals as an alternative is feasible, and without significant drawbacks for anopheline production.

## Figures and Tables

**Table 1:** Mean frequency of *Anopheles darlingi* and *Anopheles deaneorum* mosquitoes according to Sella stages.

Mosquito Species	Sella Stage	24 h		48 h		72 h		96 h	
		Honey (±SD)	Sugar (±SD)	Honey (±SD)	Sugar (±SD)	Honey (±SD)	Sugar (±SD)	Honey (±SD)	Sugar (±SD)
*Anopheles darlingi*	Stage 2	3.7 (6.4)	3.5 (6.0)	-	-	-	-	-	-
	Stage 3	63.0 (30.0)	57.9 (41.1)	0 (0)	2.1 (3.6)	-	-	-	-
	Stage 4	33.3 (35.1)	38.6 (44.7)	4.5 (3.9)	6.3 (10.9)	0 (0)	2.4 (4.1)	-	-
	Stage 5	-	-	65.2 (9.4) *	27.0 (9.5) *	8.9 (8.4)	2.4 (4.1)	6.1 (10.5)	0 (0)
	Stage 6	-	-	30.4 (12.6)	62.5 (18.8)	15.8 (8.0)	14.7 (14.3)	8.6 (8.3)	5.6 (4.9)
	Stage 7	-	-	0 (0)	2.1 (3.6)	75.3 (15.2)	80.6 (7.9)	85.4 (13.8)	94.4 (4.9)
*Anopheles deaneorum*	Stage 2	8.6 (6.2)	1.7 (2.9)	-	-	-	-	-	-
	Stage 3	64.0 (24.6)	62.2 (26.6)	-	-	-	-	-	-
	Stage 4	25.8 (21.2)	36.1 (28.0)	7.8 (13.6)	1.9 (3.2)	-	-	-	-
	Stage 5	1.7 (2.9)	0 (0)	53.3 (16.2)	56.6 (23.8)	3.9 (3.4)	1.8 (3.0)	-	-
	Stage 6	-	-	42.5 (19.3)	28.7 (31.6)	13.4 (6.9)	11.3 (9.9)	3.7 (6.4)	4.1 (7.2)
	Stage 7	-	-	7.3 (2.8)	11.1 (9.6)	82.7 (7.0)	86.9 (11.4)	96.3 (6.4)	95.8 (7.2)

* *p*-value < 0.05.

**Table 2:** Fecundity parameters according to carbohydrate source.

Species/Parameters	Honey (±SD)	Sugar (±SD)	*p*-Value
*Anopheles darlingi*			
Engorgement rate (%).	87.4 ± 16.0	93.1 ± 8.8	0.559
Eggs/female	27.5 ± 10.2	16.4 ± 5.2	0.118
Hatching rate (%)	84.1 ± 10.2	75.9 ± 18.9	0.481
*Anopheles deaneorum*			
Engorgement rate (%)	71.7 ± 19.3	70 ± 22.3	0.909
Eggs/female	15.1 ± 10.6	21.5 ± 15.5	0.519
Hatching rate (%)	64.7 ± 11.1	64.8 ± 11.9	0.985

## Data Availability

Data is contained within the article or Supplementary Materials.

## Supplementary Materials

The following supporting information can be downloaded at: https://www.mdpi.com/article/10.3390/insects15040240/s1, Table S1: Data from each DMFA.
